# Supplementary material for: Recyclable photoresins for light-mediated additive manufacturing towards Loop 3D printing
Source: Nat Commun. 2023 Sep 7;14:5504. doi: 10.1038/s41467-023-41267-w (PMC10484940; doi:10.1038/s41467-023-41267-w)
Supplement: Supplementary file 1 — Supplementary Information [file 41467_2023_41267_MOESM1_ESM.pdf]

Recyclable photoresins for light-mediated additive manufacturing towards Loop 3D printing

Xabier Lopez de Pariza[1], Oihane Varela[1], Samantha O. Catt[2], Timothy E. Long[3], Eva Blasco[2,4], and Haritz Sardon[1]

[1]POLYMAT and Department of Polymers and Advanced Materials: Physics, Chemistry and Technology, Faculty of Chemistry, University of the Basque Country UPV/EHU, Donostia-San Sebastián 20018, Spain
[2]Ruprecht-Karls-Universität Heidelberg, Organic Chemistry Institute (OCI), 69120 Heidelberg, Germany;
[3]Arizona State University, School of Molecular Science and Biodesign Center for Sustainable Macromolecular Materials and Manufacturing, Tempe, Arizona 85281, United States.
[4] Ruprecht-Karls-Universität Heidelberg, Institute for Molecular Systems Engineering and Advanced Materials (IMSEAM), Germany, 69120 Heidelberg

Supplementary Figures:

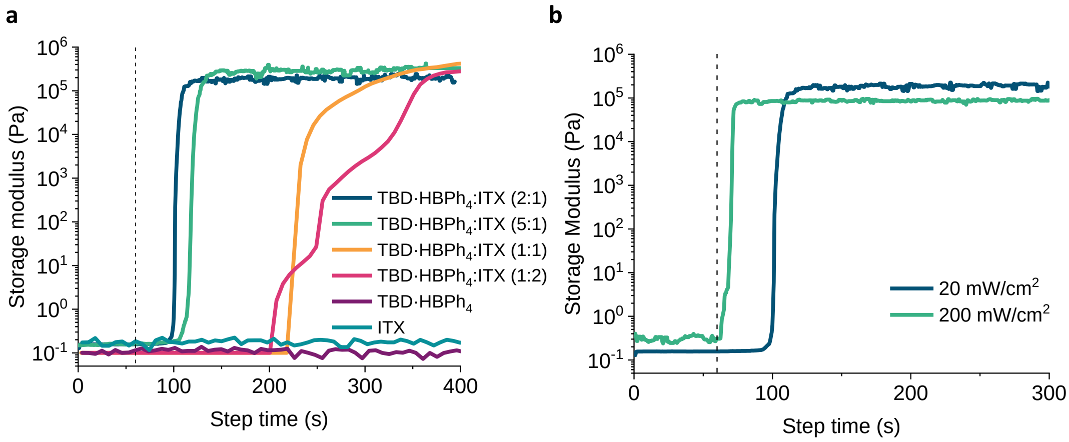

Supplementary Fig. 1: Photorheological characterization of PSU1. **a** Evolution of G' and G'' of PSU1 resin using different catalyst mixtures as function of UV-LED (365 nm) irradiation time at an intensity of 20 mW/cm^2. **b** Evolution of G' and G'' of PSU1 resin over time at different irradiation intensities.

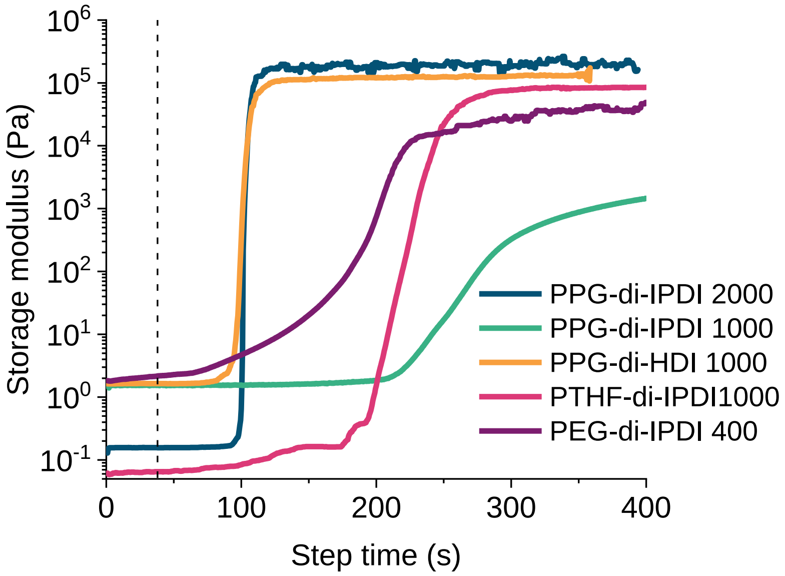

Supplementary Fig. 2: Photorheological characterization of PSU resins. Evolution of G' and G'' of for PSU resins containing different isocyanate prepolymer.

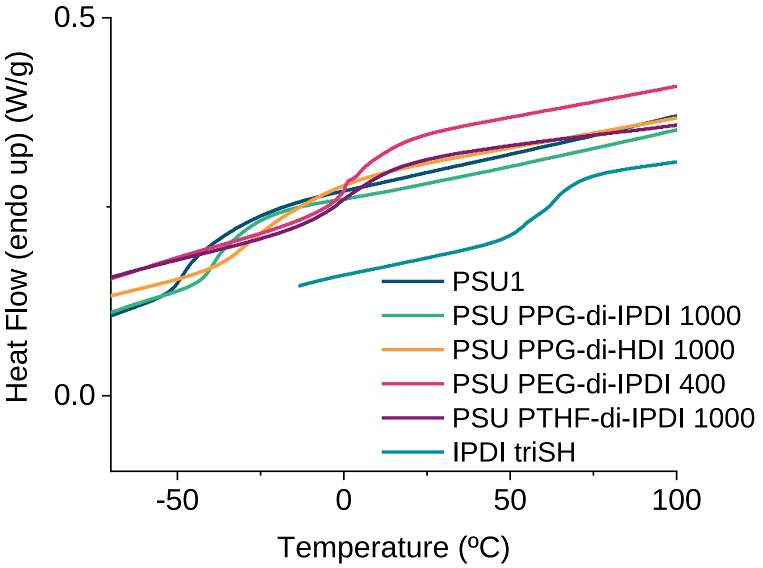

Supplementary Fig. 3: DSC traces of different PSU formulations.

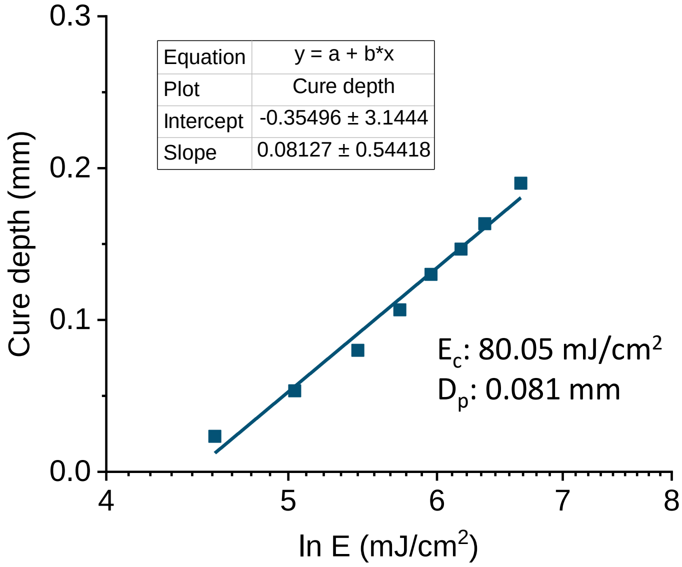

Supplementary Fig. 4: Jacob's working curve for PSU1 formulation at a constant intensity of 20 mW/cm^2.

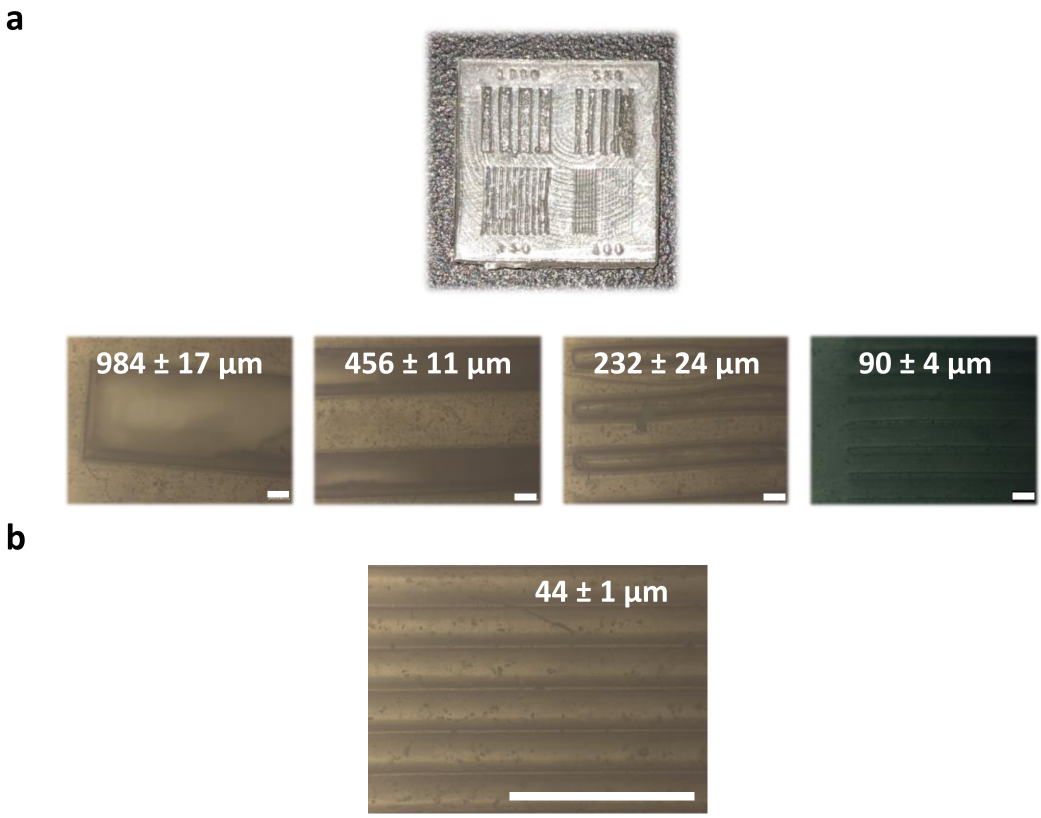

Supplementary Fig. 5: Printing resolution of DLP printed PSU1. **a** Top: Photograph of the XY resolution test 3D object with PSU1 resin. Bottom: Zoom-in microscope images of 1000 µm, 500 µm, 250 µm, and 100 µm outline shapes for resolution demonstration, the number above shows the average measured feature length. **b** Zoom-in microscope image of the Z axis of the printed sample showing the layer thickness, the number on the top of the image shows the average measured layer thickness. Scale bar 200 µm.

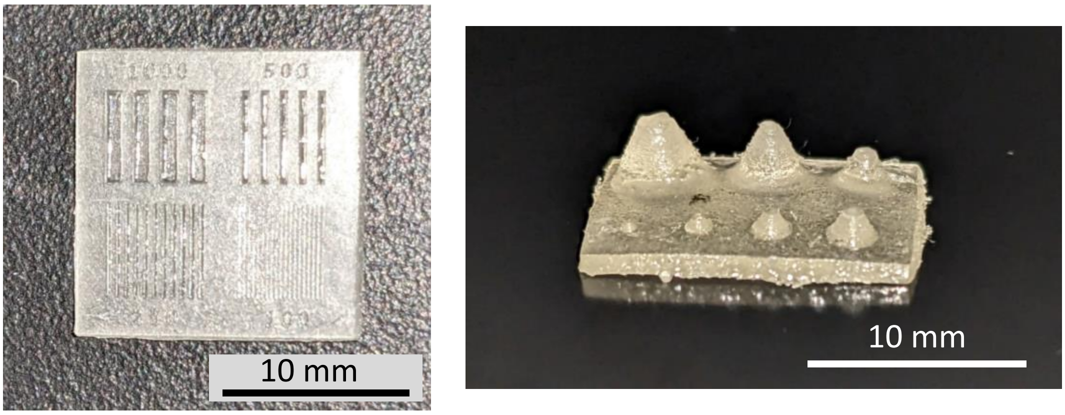

Supplementary Fig. 6: Effect of the diluent in the DLP printing resolution. Photograph of 3D printed objects printed without acetone.

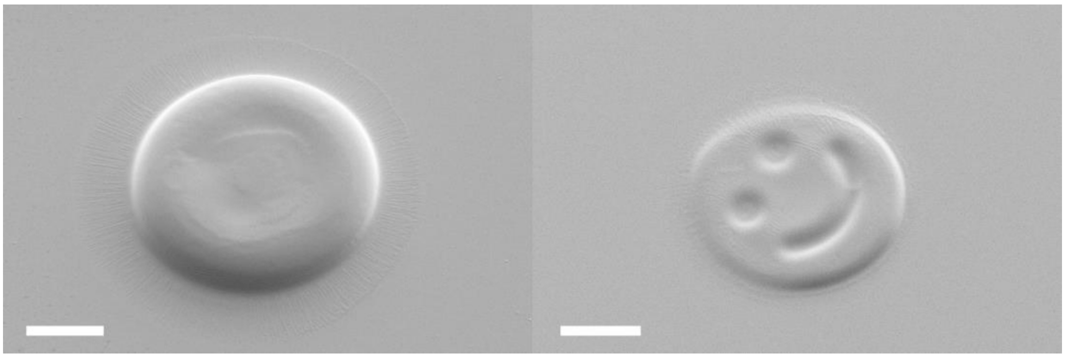

Supplementary Fig. 7: Resolution improvement by DLW when using acid in the formulation. SEM images of DLW printed smiley without octanoic acid (left) and using octanoic acid (right). Scale bar 30 µm. Original STL file has a diameter of 80 µm. The sample printed without octainoic acid has a diameter of ≈ 96 µm while the sample printed with octainoc acid has a diameter of ≈ 76 µm.

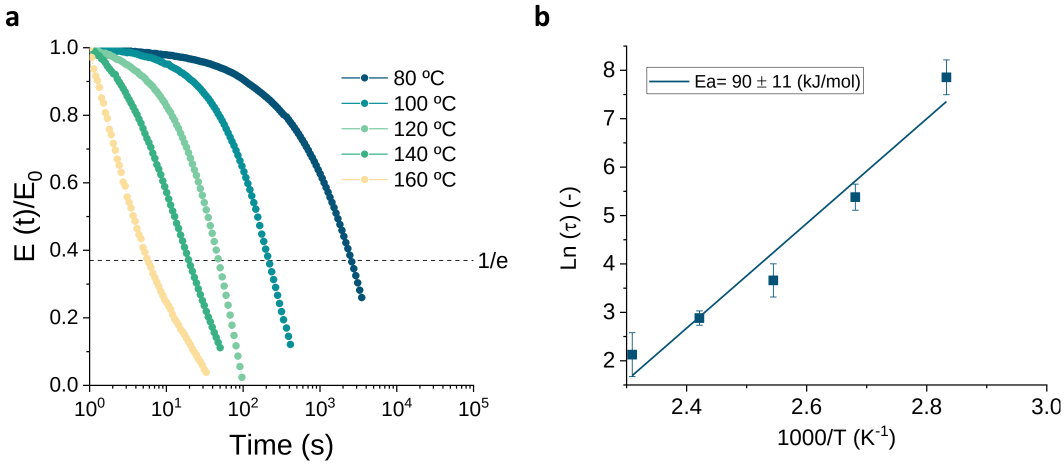

Supplementary Fig. 8: Dynamic behavior of PSU1. **a** Stress-relaxation measurements for PSU1 at different temperatures. **b** Arrhenius plots of characteristic relaxation time of PSU1 and respective activation energy (E_a). Error bars; Standard Deviation (n = 3).

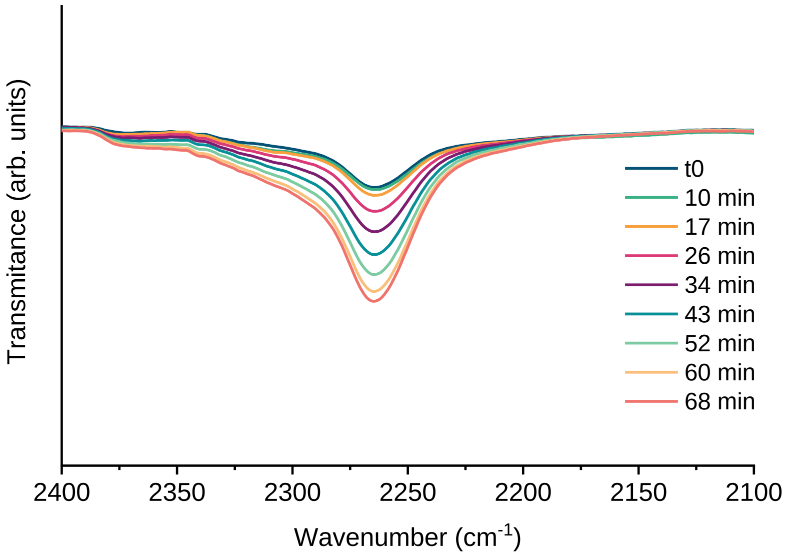

Supplementary Fig. 9: FTIR characterization of the dissociative dynamic mechanism of thiourethane bond. Time evolution of the FTIR spectra recorded at 120 ˚C of PSU1 in the region of the isocyanate (N=C=O) stretching band.

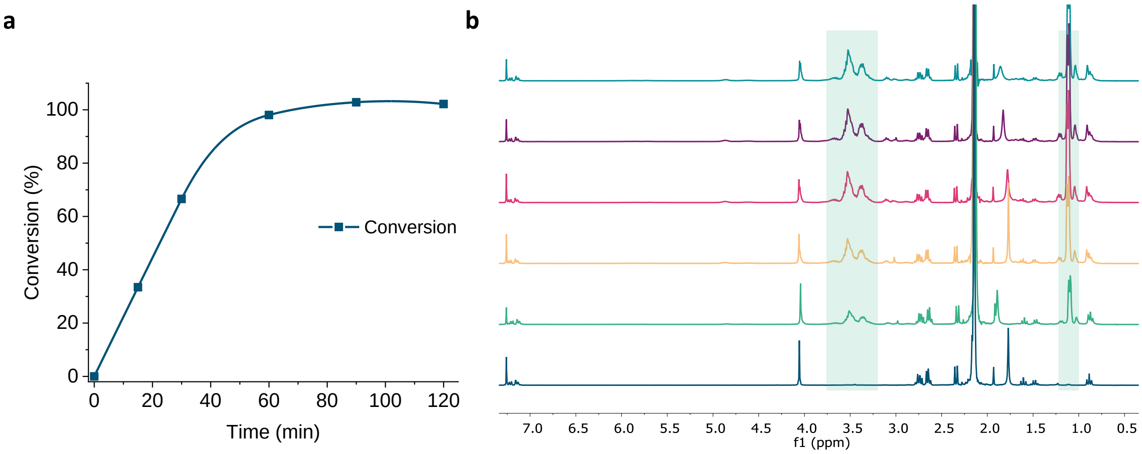

Supplementary Fig. 10: Depolymerization kinetics of PSU1. **a** Depolymerization conversion of PSU1 at the stablished conditions. **b** ^{1}H-NMR spectra at different depolymerization times. Highlighted appearance of bands attributed to the PPG containing oligomers.

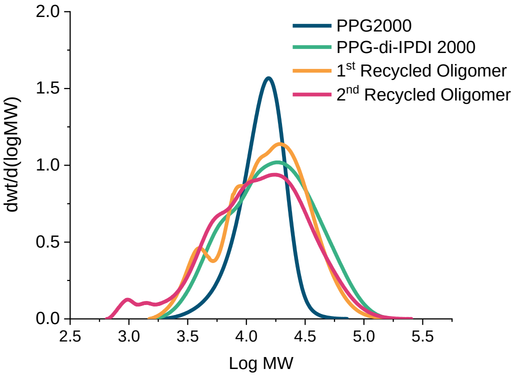

Sample	M_n (kDa)	PDI
PPG 2000	2.8	1.08
PPG-di-IPDI 2000	5.4	1.41
1st Recycled Oligomer	11.7	1.94
2nd Recycled Oligomer	7.5	2.52

Supplementary Fig. 11: SEC characterization of pristine and recycled oligomers. SEC traces for PPG 2000 (blue), PPG-di-IPDI 200 (green), 1st Recycled Oligomer (orange) and 2nd Recycled Oligomer (pink). Number averaged molecular weight (M_n) and polydispesity index (PDI) of the studied oligomers.

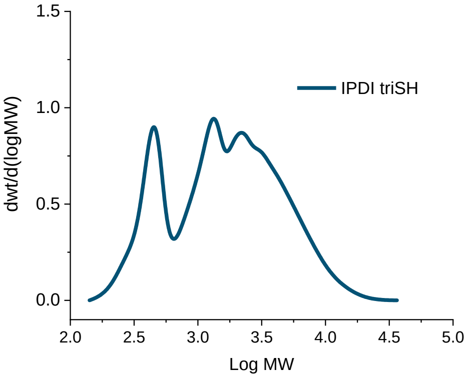

Sample	M_n (kDa)	PDI
IPDI triSH	1.0	2.56

Supplementary Fig. 12: SEC trace of IPDI triSH recycled oligomer mixture. Number averaged molecular weight (M_n) and polydispesity index (PDI).

Supplementary Tab. 1: Thiol content of recycled oligomers.

Sample	SH mol / g
1st Recycled	0.75 ± 0.02
2nd Recycled	0.70 ± 0.03

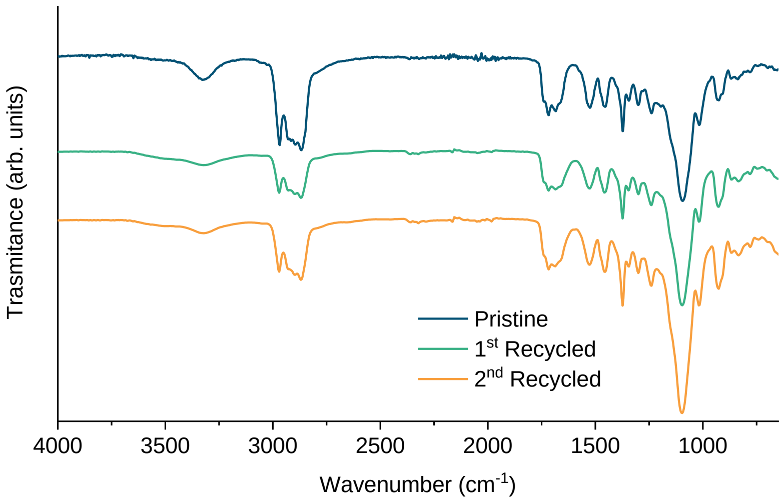

Supplementary Fig. 13: FTIR Characterization of recycled PSU materials. FTIR spectra of pristine (blue), 1st (green) and 2nd (orange) recycled PSU material.

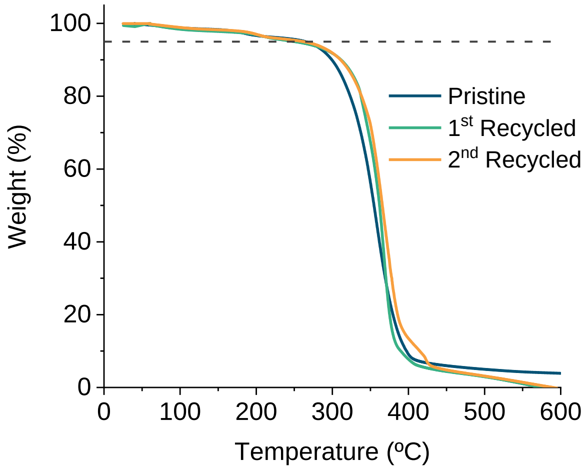

Sample	$T_{5\%}$ (˚C)
Pristine	265
1st Recycled	250
2nd Recycled	261

Supplementary Fig. 14: Thermogravimetric analysis (TGA) of PSU materials. Pristine (blue), 1st recycled (green) and 2nd recycled (orange) materials. Dashed line determines de 5 % of weight loss. In the table the onset temperatures ($T_{5\%}$).

Supplementary Tab. 2: Gel content values for PSU printed materials.

Sample	Gel content (%)
Pristine	97 ± 3
1st Recycled	95 ± 3
2nd Recycled	97 ± 2

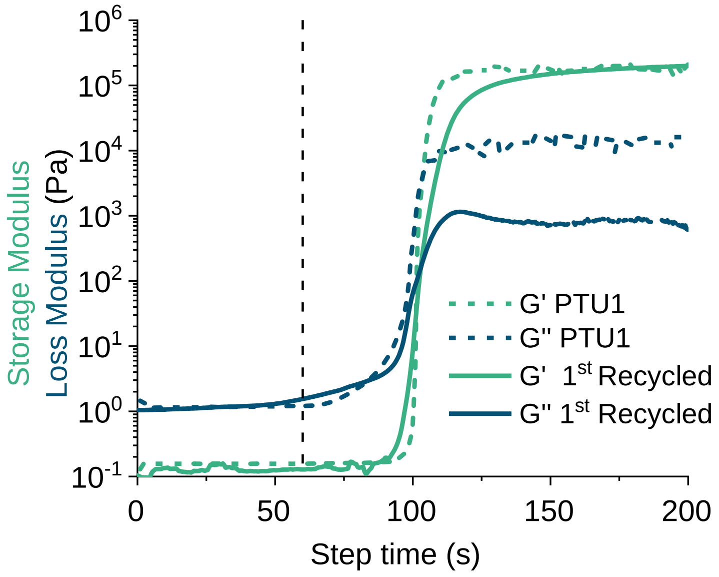

Supplementary Fig. 15: Photorheological characterization of pristine and recycled PSU1 resin. Evolution of storage modulus (G') (green) and loss modulus (G'') (blue) as function of UV-LED (385 nm) irradiation time at an intensity of 20 mW/cm². Dashed lines PSU1 resin, solid line recycled PSU1 resin.

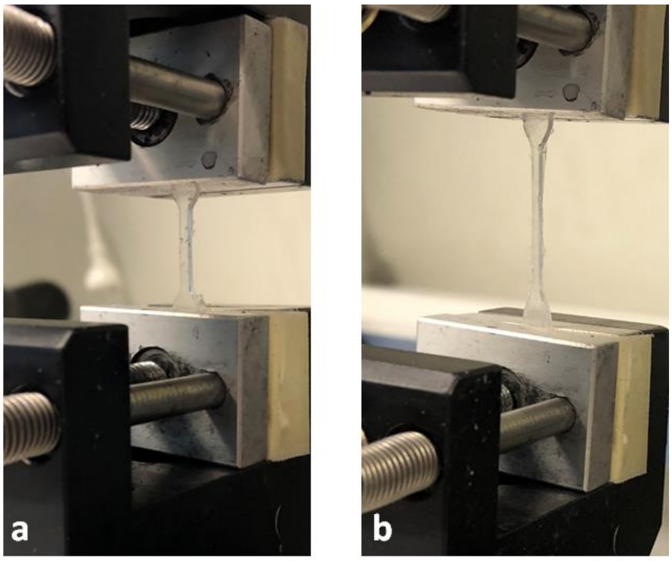

Supplementary Fig. 16: Left: Photographs of printed samples on the uniaxial tensile test equipment; **a** before and **b** during measurement.

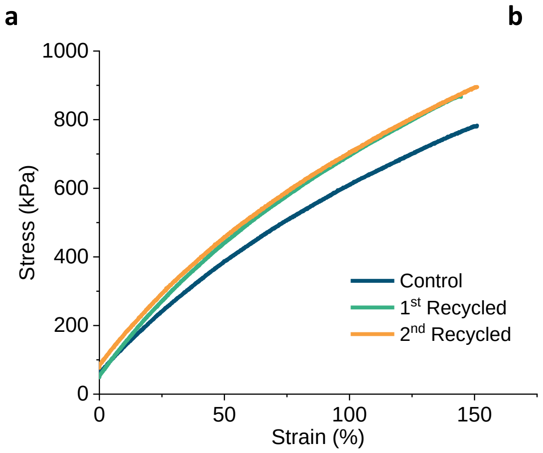

b

Sample	Deformation at break (%)	Stress at break (kPa)	Young Modulus (kPa)
Control	155 ± 5	780 ± 20	0.82 ± 0.04
1st Recycled	148 ± 10	848 ± 34	0.83 ± 0.09
2nd Recycled	146 ± 9	841 ± 44	0.77 ± 0.03

Supplementary Fig. 17: Mechanical property characterization of pristine and recycled PSU1 materials. **a** Uniaxial stress–strain curves for the pristine material (blue), 1st recycled material (green) and 2nd recycled material (orange). **b** Table summarizing the most relevant mechanical properties of the materials.

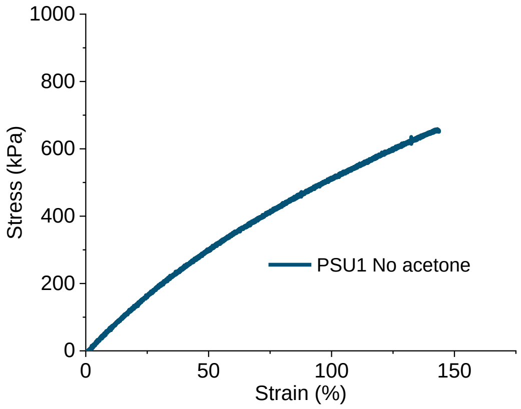

Sample	Deformation at break (%)	Stress at break (kPa)	Young Modulus (kPa)
PSU1 No acetone	145 ± 6	658 ± 11	0.66 ± 0.04

Supplementary Fig. 18: Uniaxial stress–strain curves for PSU1 printed without acetone. Right: Table summarizing the most relevant mechanical properties of the material.

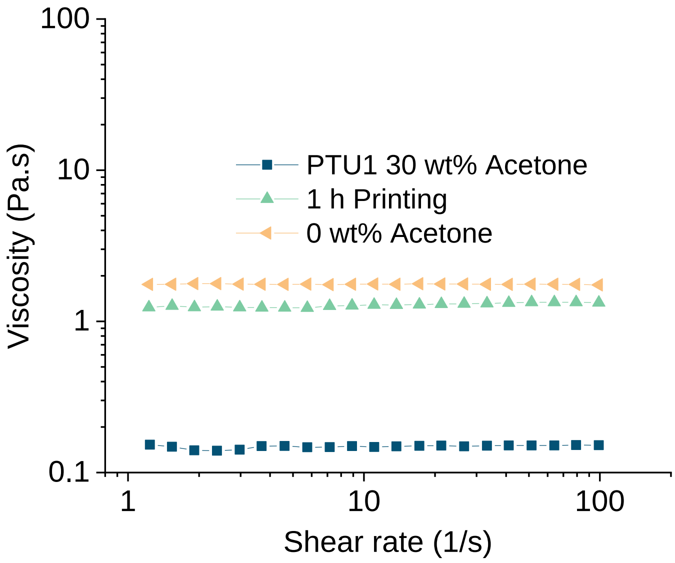

Supplementary Fig. 19: Viscosity curves of the PSU1 resin formulation (blue), PSU1 resin formulation after 1 h printing (green), and the resin formulation without acetone (orange).

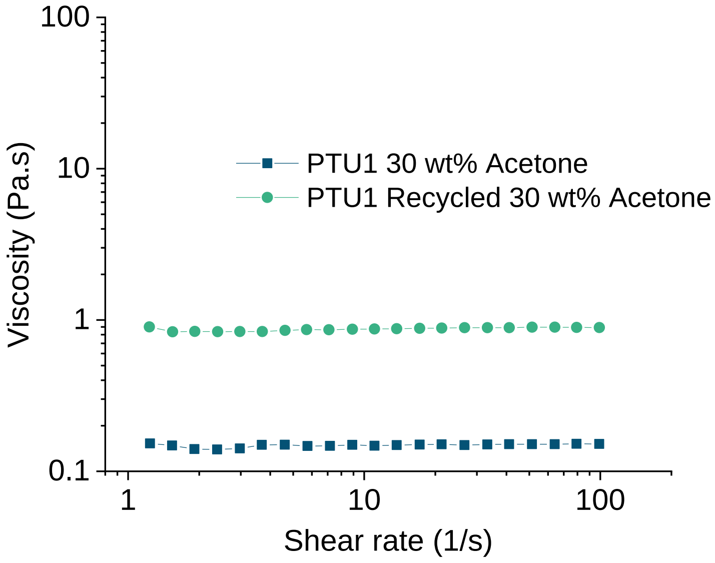

Supplementary Fig. 20: Viscosity curves of the PSU1 resin formulation (blue) and recycled PSU1 formulation (green), both containing 30 wt% of acetone as diluent.
